# Deep radiomic model based on the sphere–shell partition for predicting treatment response to chemotherapy in lung cancer

**DOI:** 10.1016/j.tranon.2023.101719

**Published:** 2023-06-13

**Authors:** Runsheng Chang, Shouliang Qi, Yanan Wu, Yong Yue, Xiaoye Zhang, Yubao Guan, Wei Qian

**Affiliations:** a College of Medicine and Biological Information Engineering, Northeastern University, Shenyang, China; b Key Laboratory of Intelligent Computing in Medical Image, Ministry of Education, Northeastern University, Shenyang, China; c Department of Radiology, Shengjing Hospital of China Medical University, Shenyang, China; d Department of Oncology, Shengjing Hospital of China Medical University, Shenyang, China; e Department of Radiology, The Fifth Affiliated Hospital of Guangzhou Medical University, Guangzhou, China

**Keywords:** Non-small cell lung cancer, Treatment response to chemotherapy, Sphere–shell partition, Radiomics, Deep learning

## Abstract

**Background:**

The prognosis of chemotherapy is important in clinical decision-making for non-small cell lung cancer (NSCLC) patients.

**Objectives:**

To develop a model for predicting treatment response to chemotherapy in NSCLC patients from pre-chemotherapy CT images.

**Materials and Methods:**

This retrospective multicenter study enrolled 485 patients with NSCLC who received chemotherapy alone as a first-line treatment. Two integrated models were developed using radiomic and deep-learning-based features. First, we partitioned pre-chemotherapy CT images into spheres and shells with different radii around the tumor (0–3, 3–6, 6–9, 9–12, 12–15 mm) containing intratumoral and peritumoral regions. Second, we extracted radiomic and deep-learning-based features from each partition. Third, using radiomic features, five sphere–shell models, one feature fusion model, and one image fusion model were developed. Finally, the model with the best performance was validated in two cohorts.

**Results:**

Among the five partitions, the model of 9–12 mm achieved the highest area under the curve (AUC) of 0.87 (95% confidence interval: 0.77–0.94). The AUC was 0.94 (0.85–0.98) for the feature fusion model and 0.91 (0.82–0.97) for the image fusion model. For the model integrating radiomic and deep-learning-based features, the AUC was 0.96 (0.88–0.99) for the feature fusion method and 0.94 (0.85–0.98) for the image fusion method. The best-performing model had an AUC of 0.91 (0.81–0.97) and 0.89 (0.79–0.93) in two validation sets, respectively.

**Conclusions:**

This integrated model can predict the response to chemotherapy in NSCLC patients and assist physicians in clinical decision-making.

## Highlights

• Predict treatment response to chemotherapy in lung cancer by using CT images.
• Partition CT images into spheres and shells of different radii around the tumor.
• Handcrafted and deep-learning-based features from each partition are integrated.
• The deep radiomic model achieves an AUC of 0.96 and 0.91 in two datasets.
• Simple sphere–shell partition replaces challenging and contentious segmentation.

## Introduction

Lung cancer is an important leading cause of cancer-related deaths, about 1.5 million new cases yearly are diagnosed [1], and a two-year survival rate is as lower as 36% [2]. Histopathologically, the most common type of lung cancer is non-small cell lung cancer (NSCLC), accounting for 80% [2], and locally advanced NSCLC accounts for about 30% of newly diagnosed cases [3,4].

In clinical practice, chemotherapy has long been the predominant first-line treatment option (single or combined regimen) [5], despite advances in various treatment options (e.g., targeted medicine and immunotherapy) that have greatly improved the prognosis of lung cancer [6]. For patients with advanced NSCLC, there is no substitute for eliminating cancer cells and prolonging patient survival with chemotherapy [7], whether as a first-line treatment regimen or as adjuvant chemotherapy with surgery, radiotherapy, targeted agents, and immunotherapy [6], [7], [8]. The prognostic response to chemotherapy varies significantly for different patients and even for different stages and phases of treatment for the same patient. Hence, predicting the treatment response to chemotherapy is essential for developing clinical treatment plans [5].

Imaging of the peritumoral region is increasingly contributing to disease screening, pathological and genotypic classification, and assessment of therapeutic responses, as the microbial environment and tissue architecture in the vicinity of the tumor may be more sensitive to clinical intervention [9,10]. The formation and spread of cancer cells are closely associated with infiltrating lymphocytes and macrophages in the tumor's vicinity [11,12]. The prognostic assessment of the radiomic features of peritumoral and intratumoral regions has been confirmed by many studies. They were used to distinguish between prostate cancer risk categories in prostate cancer patients [13] and predict early hepatocellular carcinoma after curative treatment recurrence [14].

Existing studies have relied on the segmentation of tumors, both in radiomics and deep learning. However, the current segmentation methods do not segment tumors with 100% accuracy and usually require double-blind experiments and consistency tests on multiple operators. Ashraf et al. found that the reproducibility of volumetric parameters was poor while different segmentation algorithms were used [15]. Owens et al. proposed that the intraclass correlation coefficient value varied considerably across different observers no matter whether semi-automated or manual segmentation tools were used [16].

We proposed a method to partition the region of interest (ROI) into spheres and shells from the center of the tumor based on CT images before chemotherapy. Multiple sphere-shell partitions that may contain both intratumoral and peritumoral regions were obtained. Radiomic and deep-learning-based features were extracted from these partitions. The methods of feature fusion, image fusion, and the integrated model were compared to obtain a model with the best predictive performance of treatment response to chemotherapy for NSCLC patients.

## Materials and methods

### Patients

We engaged 705 lung cancer patients who were enrolled in the Shengjing Hospital of China Medical University between July 2015 and September 2021, from which 326 NSCLC patients treated with first-line chemotherapy were selected to build up Dataset 1. The first-line chemotherapy means the first treatment regimen a patient receives after diagnosis in clinical practice is chemotherapy. And the aim of selecting the first-line chemotherapy is to eliminate interfering factors from other treatment regimens. By using the same collection criteria (Supplemental Fig. S1), 64 patients were recruited from the Fifth Affiliated Hospital of Guangzhou Medical University to form Dataset 2. Another cohort of Dataset 3 included 95 NSCLC patients enrolled between May 2022 and November 2022 in the same center as Dataset 1. Supplemental Table S1 shows the detailed CT image acquisition parameters. All cases were classified into "response" and "non-response" groups based on RECIST [17], clinical diagnostic reports, and CT images before and after chemotherapy. In contrast, the patients with complete response (CR: all target lesions disappeared) and partial response (PR: the target lesions decreased by at least 30% in the sum of the diameters) are input into the response group, and the patients with progressive disease (PD: the target lesions increased by at least 20% in the sum of the diameters) and stable disease (SD: neither sufficient shrinkage to qualify for PR nor sufficient increase to qualify for PD) are input into the non-response group. The Ethics Committee of both Shengjing Hospital of China Medical University and the Fifth Affiliated Hospital of Guangzhou Medical University approved this study. The number and date are “2018–136–2, 2018–03–08″ and “Medical Ethical Review (MER) 2017–38, 2017–04–17″ for the two approvals, respectively. Informed consent was waived because of the nature of the retrospective clinical chart review study.

### Study design

**Fig. 1:**
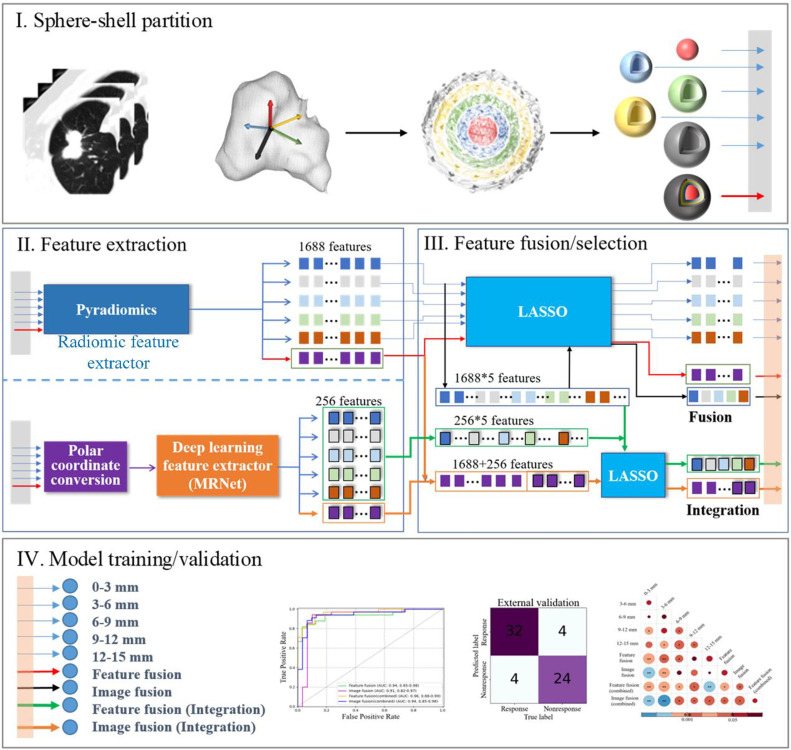
Flowchart of this research including four steps of the sphere-shell partition, feature extraction, feature fusion/integration/selection, and model training/validation.

Fig. 1 shows the flowchart of this study. There are four main steps involved in this process. First, five sphere-shell partitions were cropped by spheres of different radii according to the tumor centroids in the CT images before chemotherapy. The five partitions are combined through the way of image fusion and produce a sphere as the six partitions. Second, radiomic features and deep learning features were extracted from the above six partitions. Third, nine feature sets (five from separate sphere-shell partitions, two from image and feature fusion, and two from integration of radiomic and deep-learning features) were obtained and critical features were selected by using the least absolute shrinkage and selection operator (LASSO) method. Finally, nine models were trained and validated in Dataset 1. Finally, the best model was tested in Dataset 2 as an external validation cohort and Dataset 3 as an independent validation cohorts. The details of each step will be given in the following sections.

Dataset 1 (response: 174, non-response: 152) was randomly divided into training and independent test cohorts at a ratio of 8:2, and ten-fold cross-validation was adopted in the training cohort. The training cohort was randomly divided into ten parts; nine parts were used for training each fold, and the remaining one was used for verification. After traversing all the cohorts, the model was tested using an independent test set. Dataset 2 (response: 36, non-response: 28) and Dataset 3 (response: 44, non-response: 51) were the validation cohort to verify the model's generalization ability.

### Sphere-shell partition

We resampled all CT images into voxels of 1 × 1 × 1 mm to eliminate the influence of variables from various hospitals and acquisition devices on the prediction results. First, two radiologists (with >15 years of clinical experience) reached an agreement and manually annotated the centroids of all tumors using 3D Slicer [18]. Second, with 3 mm as the demarcation, spheres of different radii were used to crop ROIs. The specified radii range of the sphere and shells is set as 0–3, 3–6, 6–9, 9–12, and 12–15 mm based on the previous experience that the CT radiomics features from the peritumoral region of 3 mm are predictive [19]. In this study, the peritumoral regions of 1 and 2 mm have been tested and the case of 3 mm results in the best predictive performance. Based on the tumor size of the patients in this study, 5-sphere shell partitions with 3 mm as the boundary can ensure that both intra-tumor and peritumor areas are included for most subjects, and can also avoid the excessive computational effort associated with a smaller boundary. Given the good prediction of the model, this partitioning proved to be effective. Based on the Euclidean distance, we obtained each pixel point within the ROI, and the slices were cropped into the sphere–shell partitions of 0–3 mm (Sphere), 3–6 mm (Shell), 6–9 mm (Shell), 9–12 mm (Shell), and 12–15 mm (Shell). Moreover, image fusion of the above five partitions gives the sixth partition, i.e., a sphere termed “0–15 mm (Sphere)”. Each sphere–shell region might contain both intratumoral and peritumoral regions owing to the irregular shape of tumors.

### Feature extraction

(1) Radiomic features:

For each partition, PyRadiomics was used to extract 1688 radiomic features (first-order, shape, and texture) separately. Subsequently, we selected features with nonzero parameters when the mean square error was reduced to the lowest point. Five handcrafted (HC) radiomic feature vectors (HC1, HC2, HC3, HC4, and HC5) represented partitions of 0–3 mm (Sphere), 3–6 mm (Shell), 6–9 mm (Shell), 9–12 mm (Shell), and 12–15 mm (Shell), respectively.

**Fig. 2:**
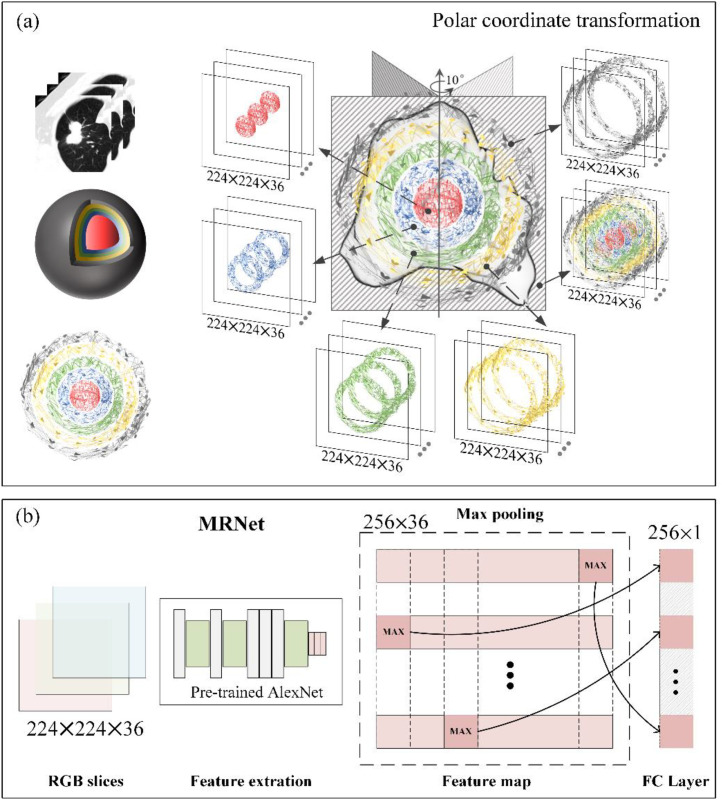
Extraction of deep-learning-based features. (a). Polar coordinate transformation (each of six sphere-shell partitions is transformed into a 224 × 224 × 36 matrix through capturing the cross-section between the partition and a plane which rotates along the z-axis at a degree of 10°); (b) Structure of MRNet (the 224 × 224 × 36 matrix is input into a pre-trained AlexNet to extract 256 × 36 deep-learning-based features and these features are merged into a feature vector of 256 elements through the Max pooling).

(1) Deep learning features:

The model used here was MRNet, which can map CT images before chemotherapy to prognostic features. As shown in Fig. 2(a), the polar coordinate transformation was first performed on the ROIs. The previously obtained sphere–shell regions were rotated every 10° along the z-axis while simultaneously taking the maximum section. We used the nearest-neighbor interpolation method. Subsequently, 36 layers of polar-coordinate CT images were available for each patient, with each layer having the same size. Each input layer of the model was a section of spherical shell on the z-axis. The feature map output from the model represents the feature combination of 36 regions of the same radius from the center of the tumor.

Training a network from scratch for image classification typically requires enormous amounts of data and time. Therefore, the weights in the AlexNet of MRNet were initialized with those pre-trained by using the ImageNet database [20], which contains 1.2 million images from 1000 categories, and then fine-tuned to fit the data for this experiment. This approach enables the first few network layers to identify standard features such as lines and edges immediately. The input size of the MRNet was 36 × 3 × 256 × 256, where 36 is the number of the CT slices and 3 is the number of channels. During the MRNet operations, each slice was passed in sequence to extract features, and a tensor of 36 × 256 × 7 × 7 was obtained through AlexNet. Then, global average pooling was applied to reduce these features to 36 × 256, and maximum pooling was used across slices to get a vector of 256 elements. This vector contains the most critical features of each layer in the CT images based on 256 kernel functions (Fig. 2(b)) [21]. Five deep learning (DL) feature vectors (DL1, DL2, DL3, DL4, and DL5) represented partitions of 0–3 mm, 3–6 mm, 6–9 mm, 9–12 mm, and 12–15 mm, respectively. MRNet was trained in the environment of Python 3.9 and PyTorch 1.10.

### Feature fusion/integration/selection

Feature fusion indicates the way of combining multiple existing feature sets before feature selection. The advantage is that the most discriminative information can be obtained from multiple original feature sets involved in the fusion. It can also eliminate redundant information because different feature sets might be correlated [22,23]. The features obtained from different partitions were combined and selected using LASSO. That is, radiomic features (5 × 1688, HC1 to HC5) from the five regions were combined and discriminative features remained. The procedure of feature fusion is presented by the black arrows in Fig. 1.

Image fusion takes all partitions (one sphere and four shells) as an entire region and performs feature extraction by applying LASSO [24,25]. We performed the feature extraction with a 15 mm radius sphere (0–15 mm sphere, Fig. 1). That is, 1688 radiomic features in total were obtained before feature selection. The procedure of image fusion is indicated by the red arrows in Fig. 1.

Integration indicates combining radiomic and deep learning features. In line with feature fusion, 1688 × 5 radiomic features are integrated with 256 × 5 deep learning features, and these features are input to LASSO to generate a feature set named “Feature fusion (Integration)”. In line with image fusion, 1688 radiomic features and 256 deep learning features extracted from the 0–15 mm sphere are integrated and input into LASSO to yield a feature set named “Image fusion (Integration)”. The procedure of two kinds of integration methods is indicated by the green and brown arrows in Fig. 1, respectively.

### Model training/evaluation

Based on the previous sphere-shell partition, feature extraction, and feature fusion/selection/integration, we can train nine models in three categories:

(a) By using radiomic features from one sphere and four shells, we can get five radiomic models: Sphere (0–3 mm), Shell (3–6 mm), Shell (6–9 mm), and Shell (9–12) mm.
(b) By using fusion methods, we can get two models: Feature fusion and Image fusion.
(c) By using the feature set of “Feature fusion (Integration)” and “Image fusion (Integration)”, we can obtain two integration models.

The above nine models were analyzed by using a support vector machine (SVM) model. The performance evaluation metrics for the models included accuracy, sensitivity, specificity, and F1-score. The AUC was used to evaluate model performance while providing a 95% confidence interval (CI). The receiver operating characteristic (ROC) and decision curve analysis (DCA) curves were utilized to display the model results. The Delong test was employed to do the statistical analysis results between the different models.

## Results

### Clinical features

This study's clinical characteristics included sex, age, smoking history, pathological type, and treatment course. The results of the statistical analysis of the clinical characteristics are shown in Table 1. Chi-square and two-sample t-tests were performed on the above characteristics between the response and non-response groups. There were no significant differences in sex, age, smoking history, and pathological type between the two groups (*p*>0.05) in Dataset 1. In Dataset 2, there were no significant differences in any clinical features between the two groups (*p*>0.05).

**Table 1:** Clinical characteristics of patients in threedatasets.

	Dataset 1			Dataset 2			Dataset 3		
Features	Response	Non-response	*p*	Response	Non-response	*p*	Response	Non-response	*p*
Patients, No.	174	152	–	36	28	–	44	51	–
Gender									
Male	92	86	1.242^a^	27	22	2.322^a^	36	41	1.214^a^
Female	82	66		9	6		8	10	
Age, y	64.28	66.21	0.783^b^	61.44	65.31	0.963^b^	62.01	61.39	0.787^b^
Pathology									
ADC	138	120	1.029^a^	22	19	2.056^a^	29	36	1.235^a^
SCC	36	32		14	9		15	15	
Smoking									
Ever	66	88	1.687^a^	26	21	1.878^a^	33	42	2.071^a^
Never	108	64		10	7		11	9	
Courses, (SD)	4.97 (1.18)	3.78 (2.23)	0.949^b^	3.24 (1.16)	3.56 (0.87)	0.657^a^	3.73 (2.01)	3.23 (1.98)	0.763^a^

ADC, Adenocarcinoma; SCC, Squamous cell carcinoma; SD, Standard deviation.

a the chi-square test.

b the two-sample *t*-test.

### Models of the different sphere–shell partitions

**Fig. 3:**
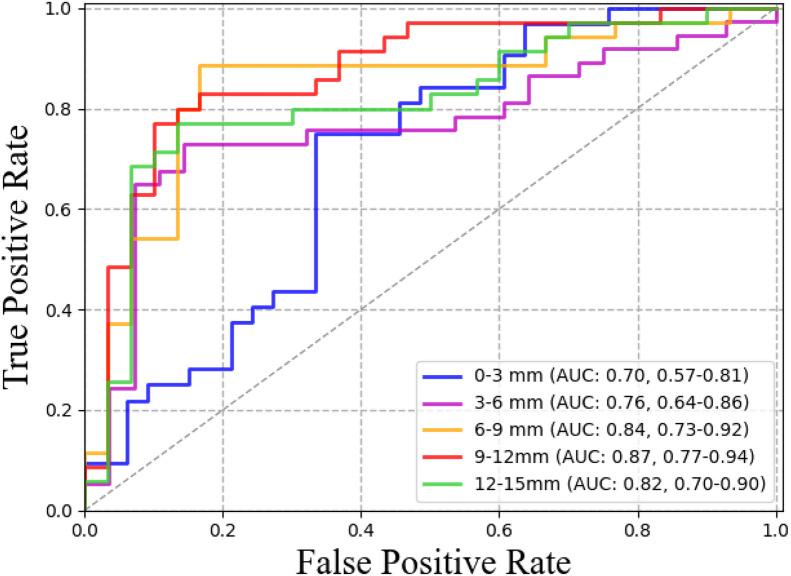
ROC of models with features extracted from different sphere-shell partitions (the highest AUC and the value at 95% confidence index: 0.87, 0.77–0.94; the model of 9–12 mm).

**Fig. 4:**
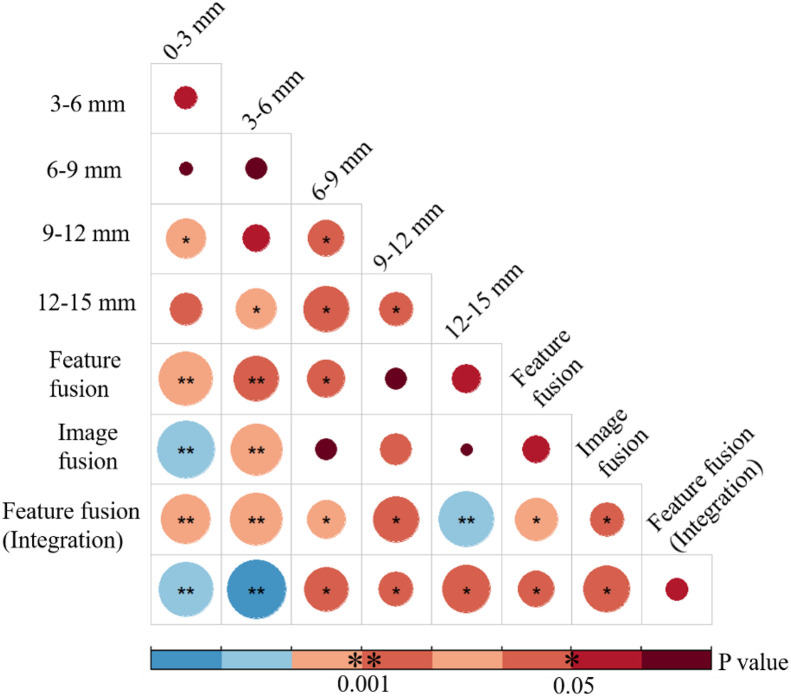
Results of the Delong test in different models (the size of the circle is correlated with the p-value of the Delong test and the color also indicates the p-value as given in the legend).

The prediction results of the different partitions obtained by SVM in the independent validation cohort are shown in Fig. 3. The highest AUC was 0.87 (95% CI: 0.77–0.94) for the 9–12 mm region. The model was correctly validated for 25 of the 35 response group and 26 of the 30 non-response group. The AUCs were 0.70 (95% CI: 0.57–0.81), 0.76 (95% CI: 0.64–0.86), 0.84 (95% CI: 0.73–0.92), and 0.82 (95% CI: 0.70–0.90) for 0–3, 3–6, 6–9, and 12–15 mm regions, respectively. The statistical results of the differences in the ROC curves of these models are presented in Fig. 4. The AUC of the 9–12 mm model was significantly higher than the 0–3 mm and 3–6 mm models (Delong test, *p*<0.05). Meanwhile, the 9–12 mm model showed a higher AUC than the 6–9 mm and 12–15 mm models, but no significant differences were observed (Delong test, *p*>0.05).

The performance metrics of the models are presented in Table 2. It is found that, for the 0–3 mm model, the accuracy, sensitivity, specificity, and F1-score were 0.71, 0.74, 0.67, and 0.73, respectively, with a cutoff value of 0.42. For the 3–6 mm model, these metrics were 0.78, 0.71, 0.87, and 0.78, respectively, with a cutoff value of 0.57. The 6–9 mm model metrics were 0.86, 0.89, 0.83, and 0.87, with a cutoff value of 0.49. For the 9–12 mm model, they were 0.83, 0.77, 0.90, and 0.83, with a cutoff value of 0.53. For the 12–15 mm model, they were 0.82, 0.77, 0.87, and 0.82, with a cutoff value of 0.57.

**Table 2:** Performance of models with different partitions, fusion methods, and datasets.

Dataset	Model	AUC	Accuracy	Sensitivity	Specificity	F1-score
Dataset 1	Sphere (0–3 mm)	0.70	0.71	0.74	0.67	0.73
	Shell (3–6 mm)	0.76	0.78	0.71	0.87	0.78
	Shell (6–9 mm)	0.84	0.86	0.89	0.83	0.87
	Shell (9–12 mm)	0.87	0.83	0.77	0.90	0.83
	Shell (12–15 mm)	0.82	0.82	0.77	0.87	0.82
	Feature fusion	0.94	0.89	0.83	0.97	0.89
	Image fusion	0.91	0.92	0.94	0.90	0.93
	Feature fusion (Integration)	0.96	0.92	0.94	0.90	0.93
	Image fusion (Integration)	0.94	0.91	0.89	0.93	0.91
Dataset 2	Feature fusion (Integration)	0.91	0.88	0.89	0.86	0.89
Dataset 3	Feature fusion (Integration)	0.89	0.88	0.89	0.88	0.88

### Performance of predictive models using two different fusion methods

**Fig. 5:**
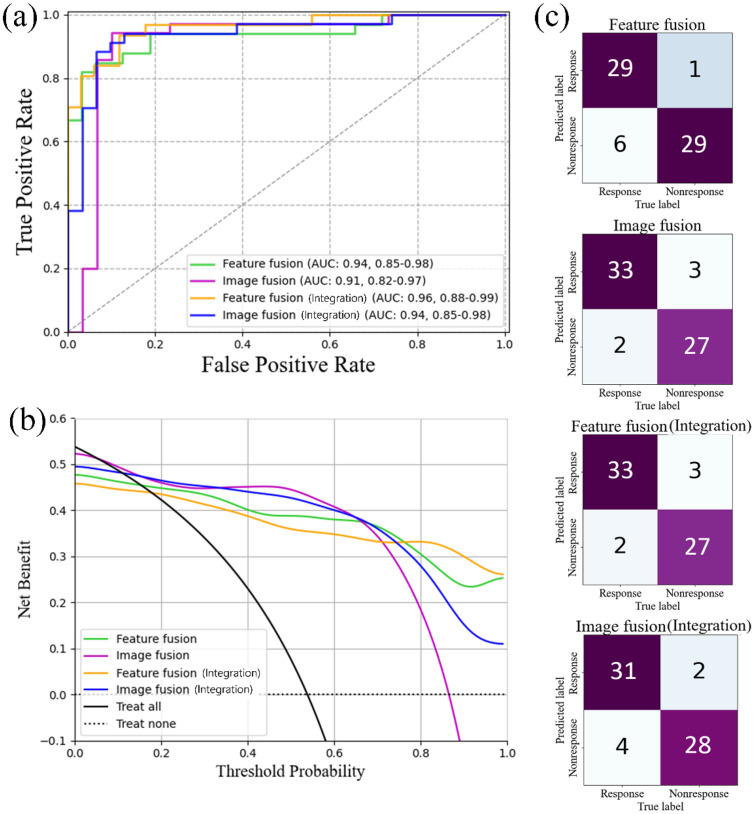
The predictive performance of different fusion methods in Dataset 1. (a) ROC curves of the four models of Feature fusion, Image fusion, Feature fusion (Integration), and Image fusion (Integration) (the highest AUC and the value at 95% confidence index: 0.96, 0.88–0.99; the model of Feature fusion (Integration)); (b) DCA curves of the developed four models and the cases of treat all and treat one; (c) Confusion matrices of the four models of Feature fusion, Image fusion, Feature fusion (Integration), and Image fusion (Integration).

For the radiomic model, feature fusion and image fusion methods were applied separately for all partitions. The predicted results for the external independent validation cohort are shown in Fig. 5. The AUC of the feature fusion method was 0.94 (95% CI: 0.85–0.98), higher than that of the image fusion method of 0.91 (95% CI: 0.82–0.97). As seen from the ROC difference statistics in Fig. 4, the AUC of the feature fusion method was higher than that of the 0–3, 3–6, and 6–9 mm models (Delong test, *p*<0.05). Higher AUCs were obtained for the 9–12, 12–15 mm, and image fusion models, but no significant differences were observed (Delong test, *p*>0.05).

The feature fusion model correctly identified 29 of 35 in the response group and 29 of 30 in the non-response group. While a cutoff value was set as 0.63, the accuracy, sensitivity, specificity, and F1-score were 0.89, 0.83, 0.97, and 0.89, respectively. The image fusion model correctly identified 33 of 35 in the response group and 27 of 30 in the non-response group and achieved an accuracy of 0.92, sensitivity of 0.94, specificity of 0.90, and F1-score of 0.93 (with a cutoff value of 0.47). The results are presented in Table 2.

### Performance of integrated models

Fig. 5 shows the predicted results of the integrated models in the independent validation cohort. The AUC of the feature fusion method was 0.96 (95% CI: 0.88–0.99), which was higher than that of the image fusion method, 0.94 (95% CI: 0.85–0.98). Out of the nine models, this model yielded the highest AUC. From the statistical results of the ROC difference in Fig. 4, the feature fusion method of the integrated model had a higher AUC than the 0–3, 3–6, 6–9, 9–12, and 12–15 mm models and the radiomic fusion model. Among the above models, there were significant differences (Delong test, *p*<0.05); however, there was no significant difference with the integrated model of the image fusion method (Delong test, *p*>0.05).

Table 2 shows that the integrated model with the feature fusion method correctly identified 33 of 35 in the response group and 27 of 30 in the non-response group, with an accuracy, sensitivity, specificity, and F1-score of 0.92, 0.94, 0.90, and 0.93, respectively, with a cutoff value of 0.34. The integrated model with the image fusion method correctly identified 31 of the 35 response groups and 28 of the 30 non-response groups. An accuracy of 0.91, sensitivity of 0.89, specificity of 0.93, and F1-score of 0.91 were achieved, while a cutoff value was set as 0.41. The performance of the above models was further analyzed by using the DCA (Fig. 5(b)). The integrated model with the feature fusion method had the largest area on the two threshold curves.

The six features applied by the integrated model with the best prediction results included two radiomic features and four deep-learning features. Run entropy (RE) (9–12 mm) showed low expression in the response group relative to the non-response group. Large dependence emphasis (LDE) (6–9 mm) showed high expression in the response group relative to the non-response group.

### External and independent validation

Dataset 2 collected from another hospital and Dataset 3 collected from another year were utilized to validate the generalization ability and robustness of the model and the results are given in Fig. 4. For Dataset 2, the integrated model had an AUC of 0.91 (95% CI: 0.81–0.97) and correctly identified 32 of 36 in the response group and 24 of 28 in the non-response group. The accuracy was 0.88, the sensitivity was 0.89, the specificity was 0.86, and F1-score was 0.89, while the cutoff value was 0.39 (Table 2). For Dataset 3, the integrated model had an AUC of 0.89 (95% CI: 0.79–0.93) and correctly identified 39 of 44 in the response group and 45 of 51 in the non-response group. The accuracy was 0.88, the sensitivity was 0.89, the specificity was 0.88, and F1-score was 0.88, while the cutoff value was 0.45 (Table 2).

## Discussion

This study aimed to predict treatment response to chemotherapy in NSCLC patients by integrating radiomic and deep learning features. We proposed asphere–shell partitioning method as an alternative for accurately segmenting the tumor itself. Using spheres of different radii, we obtained multiple sphere–shell partitions starting from the tumor centroid, including intratumoral and peritumoral regions. Then, we obtained the optimal model for predicting treatment response to chemotherapy by comparing the feature fusion, image fusion, and integrated models. The results showed that the integrated model with the feature fusion method had the best prediction ability, with an AUC of 0.96 (0.88–0.99) and an AUC of 0.91 (0.81–0.97) in the external validation cohort. This result indicated that the model had an excellent generalization ability and could effectively assist in clinical applications.

In this study, a method for applying sphere–shell partitions of different radii through the center of the tumor centroid was adopted. This method avoids the uncertainties associated with tumor segmentation, intra-group inconsistency, and manual correction of different tumor segments to form confounding factors. Even if the manually labeled centroid is not the exact geometric center of the tumor, multiple sphere–shell radii can encompass the entire tumor area, including the peritumoral region. We then aggregated the information from all regions and applied it to the final prediction through feature fusion and integrated learning methods. For clinicians, this approach is more easily reproducible and helps physicians to easily complete relevant studies. Moreover, compared to the traditional tumor segmentation methods, this method is more generalizable and easier to operate. If the average diameter of the included patient tumors is larger, this method still works by increasing the number of spherical shells (e.g., overlaying a 15–18 mm ROI). Thus, this method enables the segmentation of all types and volumes of tumor areas.

This study defined five sphere–shell partitions with a 3 mm margin containing intratumoral and peritumoral regions. The mean tumor diameter of all patients with NSCLC included here was 2.14 ± 0.68 cm. This suggests that the outperformed 9–12 mm partition, including intratumoral and peritumoral regions in a large sample, might improve the model's predictive power. Because capillaries and immune cells around the tumor may be more active than those within the tumor, they may respond more severely to chemotherapy [26]. In addition, the slightly lower predicted results in the 0–3 and 3–6 mm models suggested that the calcified and substantial tissues that might be present within the tumor were not active in chemotherapeutic drugs and were not reflected in the CT images.

We also compare the results of the two fusion methods. For both the radiomic and integrating models, the feature fusion method outperformed the image fusion method because the features from different partitions were specific and complementary [24]. This complementarity between regions can eliminate possible interference between features and complement each other's strengths. A previous study has shown that a combination of intra- and peritumoral regional features could predict whether the pathology is in complete remission after neoadjuvant radiotherapy in patients with esophageal squamous cell carcinoma [25]. It has also been reported that the combination could improve the prediction of response to chemotherapy in gastric cancer [24].

The model integrating the radiomic features and deep learning features outperformed other comparable models. Radiomic features are based on a grayscale co-occurrence matrix, which can characterize various tumor features using different algorithms [27]. Deep learning features represent high-dimensional features undetectable to the human eye [28]. Models integrated with all these features contain the strengths of all features and characterize tumors more accurately from a multidimensional perspective [29,30].

**Fig. 6:**
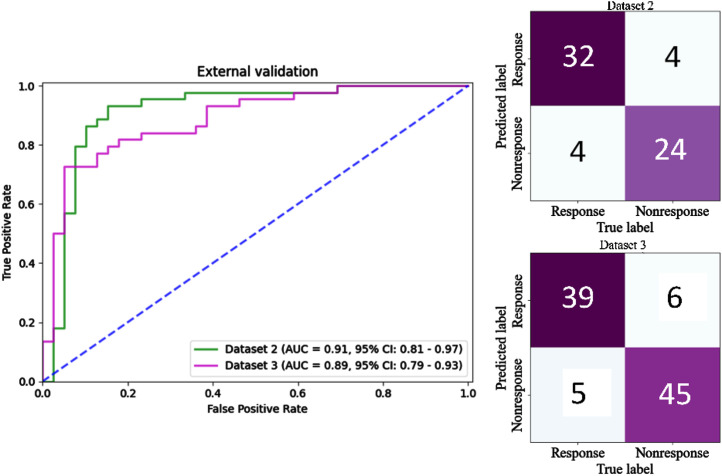
ROC curve ((AUC and the value at 95% confidence index: 0.91, 0.81–0.97)) and confusion matrix of the model (Feature fusion (Integration)) in the external validation (Dataset 2 and 3).

The RE feature showed lower expression in the response group than in the non-response group. This feature quantifies the uncertainty and randomness of the transport and gray-level distributions, with higher values indicating a higher heterogeneity of the image texture. In the response group, the LDE feature showed higher expression than in the non-response group. Moreover, this feature can quantify the large dependence distribution of the grayscale co-occurrence matrix, with larger values indicating a greater dependence, higher homogeneity, and lower heterogeneity. These two features illustrate a positive correlation between tumor heterogeneity and treatment response to chemotherapy in patients with NSCLC.(Fig. 6)

There were some limitations in this study. First, the sample size is still small. For better model training, the amount of data should be increased. Second, only one machine-learning-based classifier (i.e., SVM) was used. More deep-learning models should be used to improve the model's predictive performance. Finally, different chemotherapy drugs and doses were not considered, and they could be used as clinical features to further improve the model's predictive power.

## Conclusion

Based on sphere shells with different radii to delineate the ROIs and integration of radiomic and deep-learning-based features, the developed deep radiomic model predicted the treatment response to chemotherapy in NSCLC patients accurately. By comparing different partitioning models with feature fusion, image fusion, and integrated models, it was found that the integrated model with the feature fusion method achieved the best prediction results. This model had an excellent predictive ability and a strong generalization ability and could be applied to personalized chemotherapy regimens for clinical NSCLC patients.

## CRediT authorship contribution statement

**Runsheng Chang:** Methodology, Software, Investigation, Writing – original draft. **Shouliang Qi:** Conceptualization, Supervision, Writing – review & editing, Resources, Funding acquisition. **Yanan Wu:** Data curation, Formal analysis. **Yong Yue:** Data curation, Supervision. **Xiaoye Zhang:** Writing – review & editing, Resources. **Yubao Guan:** Data curation, Supervision. **Wei Qian:** Supervision, Funding acquisition.

## Declaration of Competing Interest

The authors declare that they have no known competing financial interests or personal relationships that could have appeared to influence the work reported in this paper.

## Appendix Supplementary materials

Image, application 1
