# Supplementary material for: Deep radiomic model based on the sphere–shell partition for predicting treatment response to chemotherapy in lung cancer
Source: Transl Oncol. 2023 Jun 13;35:101719. doi: 10.1016/j.tranon.2023.101719 (PMC10277572; doi:10.1016/j.tranon.2023.101719)

Supplementary Material for

## A deep radiomic model based on sphere-shell partition for predicting chemotherapy response in lung cancer

## Table S1. Parameters for CT image acquisition

| Parameter | Dataset 1 | Dataset 2 |
| --- | --- | --- |
| Voltage [kV] | 120 | 120 |
| Tube current [mA] | 229.02 | 273.14 |
| Slice thickness [mm] | 2.5 (*n =* 23); 3.0 (*n =* 283); 5.0 (*n =* 20) | 2.0 (n = 64) |
| Pixel size [mm] | 0.82 ± 0.06 | 0.88 ± 0.22 |
| Manufacturer | GE Medical (*n =* 12), Siemens (*n =* 16),  Toshiba (*n =* 15), Philips (*n =* 283) | Siemens (*n =* 64) |

## Fig. S1. Criteria of the data selection.


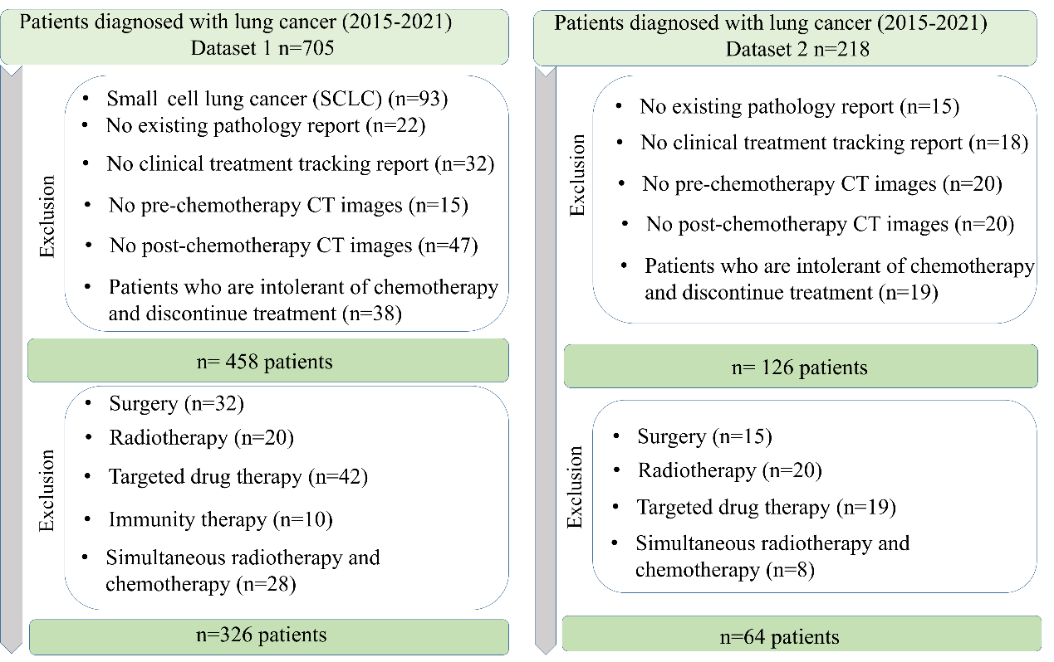

Supplement: Supplementary file 1 [file mmc1.docx]
